# Physical activity prevents alterations in mitochondrial ultrastructure and glucometabolic parameters in a high-sugar diet model

**DOI:** 10.1371/journal.pone.0172103

**Published:** 2017-02-15

**Authors:** Karina Barbosa de Queiroz, Kinulpe Honorato-Sampaio, Joamyr Victor Rossoni Júnior, Diego Andrade Leal, Angélica Barbosa G. Pinto, Lenice Kappes-Becker, Elisio Alberto Evangelista, Renata Guerra-Sá

**Affiliations:** 1 Laboratório de Bioquímica e Biologia Molecular, Núcleo de Pesquisas em Ciências Biológicas, Universidade Federal de Ouro Preto, Ouro Preto, Minas Gerais, Brasil; 2 Faculdade de Medicina, Campus JK, Universidade Federal dos Vales Jequitinhonha e Mucuri, Diamantina, Minas Gerais, Brasil; 3 Laboratório de Bioquímica Metabólica, Núcleo de Pesquisas em Ciências Biológicas, Universidade Federal de Ouro Preto, Ouro Preto, Minas Gerais, Brasil; 4 Centro de Esportes, Universidade Federal de Ouro Preto, Ouro Preto, Minas Gerais, Brasil; Universidad Pablo de Olavide, SPAIN

## Abstract

Endurance exercise is a remarkable intervention for the treatment of many diseases. Mitochondrial changes on skeletal muscle are likely important for many of the benefits provided by exercise. In this study, we aimed to evaluate the effects that a regular physical activity (swimming *without* workload) has on mitochondrial morphological alterations and glucometabolic parameters induced by a high-sugar diet (HSD). Weaned male Wistar rats fed with a standard diet or a HSD (68% carbohydrate) were subjected to 60 minutes of regular physical activity by swimming (*without* workload) for four- (20 sessions) or eight-week (40 sessions) periods. After training, animals were euthanized and the sera, adipose tissues, and skeletal muscles were collected for further analysis. The HSD increased body weight after an 8-week period; it also increased the fat pads and the adipose index, resulting in glucose intolerance and insulin resistance (IR). Transmission electron microscopy showed an increase in alterations of mitochondrial ultrastructure in the gastrocnemius muscle, as well as a decrease in superoxide dismutase (SOD) activity, and an increase in protein carbonylation. Regular physical activity partially reverted these alterations in rats fed a HSD, preventing mitochondrial morphological alterations and IR. Moreover, we observed a decrease in *Pgc1α* expression (qPCR analysis) in STD-EXE group and a less pronounced reduction in HSD-EXE group after an 8-week period. Thus, regular physical activity (swimming *without* workload) in rats fed a HSD can prevent mitochondrial dysfunction and IR, highlighting the crucial role for physical activity on metabolic homeostasis.

## Introduction

Obesity prevalence in childhood has dramatically increased in the last three decades, as well as the morbidity and mortality risks later in life [1–4]. Although the disease is considered a multifactorial disorder and it is often associated with a high-fat diet intake, excessive consumption of sugar in early life has been described as a lipogenic modulator, affecting critical periods during childhood and promoting obesity in young adults [5–8]. Related to this nutritional behaviour, current evidence indicates that a decrease in children’s daily physical activity, has contributed to the increase in obesity prevalence around the world [1–3].

It is noteworthy that physical activity induces positive adaptations for metabolic homeostasis, which could lead to significant changes in lifestyle, and could also allow us to identify molecular responses that may be useful as both therapeutic targets and for exercise prescription [9]. Besides, training adaptation in skeletal muscle is deeply influenced by a number of factors such as regularity, intensity, and diet. For instance, a regular exercise program can increase mitochondrial density and function in muscle [10–15]. Mitochondrial biogenesis is strongly induced by endurance training [16–18] and occurs via coordinated gene expression, being the expression of peroxisome proliferator-activated receptor gamma coactivator 1α (PGC1α) the critical node for signalling to mitochondrial biology, inducing mitochondrial biogenesis and oxidative capacity [19–21]. These observations have led to the widespread statement that PGC1α is required for exercise-induced mitochondrial biogenesis [18,22–24]. On the other hand, a decrease in mitochondrial content (due to reduced mitochondrial biogenesis or damaged mitochondria accumulation) can result in mitochondrial dysfunction, which hinders the mitochondrial capability to function properly [25,26].

Mitochondrial dysfunction was first described in the context of glucose intolerance 40 years ago [27]. The majority of studies in this area since that time have focused on mitochondrial changes in skeletal muscle, once obese and diabetic patients have impaired mitochondrial function in this tissue [28]. It is noteworthy that reactive oxygen species (ROS) can lead to mitochondrial dysfunction [29], as well as mitochondria can be both the primary source of ROS and the primary target of ROS damage [30,31]. Moreover, pathological conditions such as obesity may increase ROS production [32,33]. Thereby, mitochondrial dysfunction induced by ROS could result in diminished fuel oxidation, mostly fatty acids, and a consequential accumulation of by-products of lipid metabolism, comprising diacylglycerols (DAG) and ceramides (CER) [25]. It may impair mitochondrial function, activating a vicious cycle that culminates in insulin resistance (IR) [26,34].

Previous studies performed by our group have demonstrated the effects of endurance training on a treadmill on adipose tissue of weaned rats fed with a high-sugar diet (HSD) in two different periods (4-week and 8-week periods) [35, 36]. It is worth mentioning that the achievement of exercise intensity and duration ensured that a significant endurance training adaptation had been produced. At first, we showed that rats fed a HSD and subjected to endurance training for an 8-week period had an impairment in the relationship between mRNA levels of uncoupling protein 1 and 3 (*Ucp1/ Ucp3*). This change may result in lower energy efficiency and may explain the increase in the adipose index observed in these animals [35]. Thereafter, we have investigated the molecular mechanism behind weight gain in trained rats fed a HSD, and have purposed a pleiotropic effect of leptin on white adipose tissue, via its receptor *OB-Rb*. The down regulation observed in *OB-Rb* after an 8-week period of endurance exercise avoided lipolysis in retroperitoneal white adipose tissue (rWAT) and promoted triacylglycerol (TAG) synthesis and storage. As a result, we observed high levels of serum leptin and an increase in the total mass of rWAT [36].

Because of these considerations, it is noteworthy that physical activity has been of great importance on the metabolic syndrome treatment, since exercise training improves glucose tolerance and reduces IR. Furthermore, exercise training at early ages can be more effective in metabolic control due to the energetic expenditure, which impairs body weight gain and, consequently, leads to a reduced lipid storage and an improvement in oxidative balance [37, 38]. However, the adaptation to exercise depends on aspects such as training load, frequency, and duration [39]. It is remarkable that the treadmill endurance training in our previous works was not able to reduce the HSD-induced metabolic impairment [35,36], pointing to a crucial role of the training type to set exercise benefits in obese and diabetic patients, including children. Moreover, the effects of a regular physical activity program (*without* a workload) on rats fed a HSD have not been elucidated yet.

Considering that skeletal muscle is an important tissue in metabolic control and is very modified by exercise training, in this study we aimed to elucidate the mitochondrial and glucometabolic changes induced by regular physical activity (swimming *without* workload) in rats fed a HSD. We hypothesized that regular physical activity may induce mitochondrial biogenesis via *Pgc1α* expression, preventing alterations in mitochondrial ultrastructure, thus improving the IR induced by a HSD. According to our results, regular physical activity has a protective effect in obese animals fed a HSD, up regulating *Pgc1α* expression and preventing mitochondrial dysfunction and IR after an 8-week period.

## Material and methods

### Animals and diet

All of the experimental procedures were authorised by the Ethical Committee for Animal Care of the Federal University of Ouro Preto (Protocol 099/2013) and were conducted in accordance with the regulations described in the Committee’s Guiding Principles Manual.

Four-week-old weaned male Wistar rats (60–65 grams) were bred at Centro de Ciência Animal (CCA, Federal University of Ouro Preto, Ouro Preto, MG, Brazil), housed in individual cages under controlled light (5–19 hours) [35, 36] and temperature (24±2°C) conditions; water and rat chow were provided *ad libitum*. The animals in the experimental groups were fed a HSD (68% carbohydrates) consisting of 33% standard chow (Nuvilab CR1, Colombo, Brazil), 33% condensed milk, and 7% sucrose by weight (the remainder percentage consisted of water) for 4-week and 8-week periods [40]. The control groups were fed only standard chow (STD). The composition of each diet has been previously published [35].

Rats had their calorie intake and body weight measured once a week during the experimental period. After 4-week and 8-week periods, the weekly food intake was multiplied by the energy density for the STD (12.22 kJ/g) and the HSD (13.31 kJ/g), to calculate the energy intake.

### Experimental design

Before the beginning of the experimental procedures, 96 male Wistar rats were randomly divided into the following four groups: 1) sedentary rats fed a standard chow (STD-SED, standard diet–sedentary; N = 24), 2) swimming rats fed a standard chow (STD-EXE, standard diet–exercise; N = 24), 3) sedentary rats fed a high-sugar diet (HSD-SED, high-sugar diet–sedentary; N = 24), and 4) swimming rats fed a high-sugar diet (HSD-EXE, high-sugar diet–exercise; N = 24). Thereafter, the four groups were divided into two different swimming periods: 4-week (N = 12) and 8-week periods (N = 12). Physical activity started at the same time as the introduction of the HSD. At the end of their respective swimming periods, the animals in each group were euthanized by decapitation and the sera, adipose tissues (retroperitoneal, epididymal, and inguinal), and skeletal muscles (gastrocnemius and soleus) were collected. The gastrocnemius muscle was chosen to assess mitochondrial and oxidative damage due to its fibre-type, richness in glycolytic fibres, and responsiveness to diet-effects [41], while the soleus was used to measure the oxidative capacity induced by regular physical activity [39]. To evaluate the development of obesity, the adiposity index was calculated with the following equation: 100 × [sum of fat pad weights (g)/ body weight (g)] [42]. To calculate the fat pad weight, we used all the white adipose tissues that were removed and they were represented as relative weight: [fat pad weight (g)/ body weight (g)] × 100. The physical activity protocol was carried out without a workload.

### Regular physical activity protocol

The physical activity protocol was performed in a clear glass swimming pool (175 cm × 53 cm × 65 cm), in which they could see outside, the other rats swimming, and could orient themselves to their environment. Each rat swam in an individualized compartment, avoiding climbing behaviour, and in warm water (between 30 and 32°C), as previously published [39]. After each session of swimming, the rats were dried to prevent decreases in body temperature.

The regular exercise began with an adaptation period. The first training session lasted 2 minutes, and increased daily (10, 20, and 40 minutes) until reaching 60 minutes on the fifth day of the adaptation week. After this period, exercised groups carried out swim sessions of 60 minutes, five times per week, during 4-week (20 swimming sessions) or 8-week periods (40 swimming sessions). Non-continuous behaviour, like floating or diving, was avoided [43]. Sedentary groups underwent 2 minutes sessions during the experimental period. The animals were euthanized 24 hours after completion of the swimming protocol, in a fasted state.

### Sera parameters and Oral Glucose Tolerance Test (OGTT)

Plasma glucose (N = 12) levels were measured using a Random Access Clinical Analyser (Wiener Lab, CM 200, São Paulo, Brazil) with Labtest kit (Labtest Diagnóstica SA, Minas Gerais, Brazil). Serum insulin concentration (N = 12) was assayed using a rat insulin ELISA (Millipore, São Paulo, Brazil).

The OGTT was performed in fasted rats (12 hours). Glucose levels from tail blood samples were monitored at 0, 30, 60, 90, and 120 min after gavage, using an Accu-Check glucometer (Accu-Chek Active, Roche Diagnostics Corp, Hague Road, IN), according to a previously published protocol [44]. The data were presented as the area under the curve (AUC). Finally, we calculated an insulin resistance index (HOMA) using the formula: (fasting serum insulin × plasma glucose)/ 22.5 [45].

### Citrate synthase assay

The biomarker of oxidative metabolism in soleus muscle tissues was measured using a Citrate Synthase Assay kit (Sigma-Aldrich, St. Louis, MI), using a previously published protocol [35]. Briefly, tissue samples were homogenized (50 mM Tris–HCl, 1 mM EDTA, and 0.01 mM phenylmethylsulphonyl fluoride; pH 7.4) using a Polytron homogenizer, and then the homogenates were centrifuged (725 × g for 10 min at 4°C). The supernatant was decanted, and the citrate synthase activity was assayed according to the manufacturer’s protocol.

### Mitochondrial density

For electron microscopy, posterior midbelly fragments of the gastrocnemius muscle, which are rich in white fibers [46], were dissected from three animals per group. The specimens were fixed in Karnovsky’s solution (2.5% glutaraldehyde and 2% paraformaldehyde) in 0.1 M cacodilate buffer pH 7.4 overnight at 4°C. They were post-fixed in a mixture of 2% (w/v) osmium tetroxide and 1.5% (w/v) potassium ferrocyanide for 2 hours to enhance contrast of organelles. Specimens were washed in distilled water and kept in 2% uranyl acetate (*en bloc* staining) overnight. The samples were then serially dehydrated in graded ethanol baths and embedded in Epon 812. Specimens were sectioned in 50 nm ultrathin sections and stained with Reynolds lead citrate. Transmission electron microscopy (TEM) was performed using a FEI Tecnai G2-12 Spirit at 80 kV, which exhibits a point resolution of 0.49 nm. TEM was equipped with a SIS-MegaView 3 CCD camera and acquired images showed 1373 x 1070 pixels. Twenty-five electron micrographs per animal were taken at a ×11.000 magnification. Images were randomly selected from central parts of muscle fibers and were analysed with ImageJ [47]. Volume densities (Vv) of glycogen, lipid droplets, normal, and altered mitochondria were determined with the classic point counting method using a 130-point-grid (700 x 700 nm grid) projected onto each image (S1 Fig) [48, 49]. Altered mitochondria were defined as those that showed a swollen appearance with a rarefied matrix and damaged cristae, as previously described [16, 50].

### Superoxide Dismutase (SOD) activity and protein oxidation analysis

The activity of the total antioxidant enzyme SOD was measured using a Superoxide Dismutase Assay Kit (cat# 706002; Cayman Chemical Company, Ann Arbor, MI). Briefly, 150 mg of gastrocnemius muscle samples were homogenized in cold 20 mM HEPES (pH 7.2) containing 1 mM EGTA, 210 mM mannitol, and 70 mM sucrose. Ten microliters of supernatant were used in the assay. The reaction was initiated by adding xanthine oxidase. The plate was incubated on a shaker for 20 min at 24°C, and the absorbance at 450 nm was measured using a plate reader (ELx808 Absorbance Reader, Biotek, Winooski, VT). The SOD activity was expressed as U/mL per mg of tissue.

Protein oxidation by ROS leads to the formation of carbonyl derivatives, which can be measured by sensitive methods. Measurements of carbonylated protein were performed according to the method purposed by Levine [51]. Briefly, proteins were precipitated using trichloroacetic acid (TCA) (10%) and incubated in the dark with 2,4-dinitrophenylhydrazine (DNPH) and HCl at room temperature for 30 min. TCA (10%) was added to the precipitate and centrifuged at 4700 × g for 5 min at 4°C. After discarding the supernatant, the precipitate was washed twice with ethanol/ethyl acetate (1:1 v/v) dissolved in a 6% sodium dodecyl sulphate (SDS) solution and centrifuged at 18000 × g for 10 min at 4°C. Supernatant absorbance was measured at 370 nm. Carbonyl content was calculated using the DNPH molar extinction coefficient (21 × 10^3^ mol^-1^.cm^-1^), and the results were expressed as nanomoles of carbonyl groups per mg of protein. Total protein concentrations were determined using the BCA quantification method (Sigma-Aldrich, St. Louis, MI).

### Total RNA preparation and *Pgc1α* expression analysis by qRT-PCR

Total RNA was obtained from the gastrocnemius muscle using a combination of Trizol reagent (Invitrogen, São Paulo, Brazil) and chloroform (Merck, São Paulo, Brazil) for extraction, according to the manufacturer’s protocol. Then, total RNA was purified on a column using the SV Total RNA Isolation System (Promega, São Paulo, Brazil), according to the manufacturer’s protocol. Total RNA was quantified using the NanoVue system (GE) and analysed by electrophoresis on a 1.2% agarose formamide-TBE (Tris/ Borate/ EDTA) gel. Total RNA was treated with RNase-free DNase I (Promega, São Paulo, Brazil) for 30 min, and the optical density of the solution was measured at 230, 260, and 280 nm. Ratios greater than 1.8 (260/280 and 260/230) were considered to be acceptable for gene expression quantification [52].

Two micrograms of total RNA were reverse transcribed into cDNA using the High Capacity cDNA RT kit (Applied Biosystems, São Paulo, Brazil). Then, mRNA expression was quantified by qRT-PCR using the SYBR Green system (Applied Biosystems, São Paulo, Brazil) and the ABI 7300 Real-Time PCR System was used to detect the target. A rat-specific primer was used to detect *Pgc1α* [(NM_031347.1), F-5'-GCACAACTCAGCAAGTCCTC-3' and R-5'-CCAAACAGCCGTAGACTG-3']. Cycle thresholds were determined based on the SYBR Green emission intensity during the exponential phase. Cq data were normalized by *rRNA 18S* [(X01117.1) F-5'-GTAAGTGCGGGTCATAAG-3' and R-5'-CCATCCAATCGGTAGTAGC-3'], which was stably expressed in all experimental groups. The relative gene expression was calculated using the 2^−ΔCq^ method [53].

### Statistical analysis

Statistical analyses were performed using the Graph Pad Prism (version 6.01) software (Irvine, CA, USA). The sample size was determined with a power of 0.9 and a significance level (α) of 0.05. The highest estimated size to assess our outcomes was chosen (N = 12). A Shapiro-Wilk test was used to verify data's normalization. The data are reported as the mean ± standard deviation (S.D.). Differences between groups were evaluated using two-way ANOVA followed by Bonferroni test. *P*-values < 0.05 were considered statistically significant.

## Results

### Characteristic of animals submitted to regular physical activity and a HSD

Data related to the biometrical characteristic of experimental groups are shown in Tables 1 and 2. According to our results, calorie intake did not differ among groups, even in rats fed with a HSD (Tables 1 and 2). However, body weight was increased by diet (*P*<0.05) in a 4-week period, rising in the HSD-EXE group (Table 1). The interaction between diet and regular physical activity reduced the weight gain after an 8-week period (*P*<0.001), decreasing in the STD-EXE group (as compared to the sedentary control and when compared to HSD-EXE group) (Table 2).

**Table 1:** Characteristics of rats fed a STD or HSD in a 4-week period.

	4-week period				*P*-values		
	STD-SED	HSD-SED	STD-EXE	HSD-EXE	Effect of diet	Effect of exercise	Interaction
**Calorie intake (kJ)**	329±76.9	303.5±76.9	353.6±94.6	330.8±90.2	0.5496	0.5206	0.9732
**Body weight (g)**	168.4±11.9	173.5±12	154.4±20.5	182.1±26.7*	0.0195	0.6863	0.0987
Epididymal (g)**	0.48±0.21	0.72±0.18	0.5±0.12	0.68±0.14	*P*<0.001	0.8325	0.5473
Retroperitoneal (g)**	0.2±0.08	0.5±0.07*	0.3±0.1	0.4±0.2	*P*<0.001	0.9607	0.5949
Inguinal (g)**	1.0±0.22	1.5±0.3*	0.96±0.3	1.3±0.44	*P*<0.001	0.4384	0.6987
**Adipose index**	1.7±0.4	2.6±0.4*	1.6±0.5	2.4±0.7*	*P*<0.001	0.3307	0.6713
**CS activity**	5.6±0.8	5.6±0.8	16.5±3.6 ^Ɨ^	8.76±2.4*	0.0049	*P*<0.001	0.0049

Data are expressed as means ± S.D. Statistically significant differences were determined using a two-way ANOVA to examine the effects of diet (HSD or STD) and exercise (trained or sedentary), followed by Bonferroni post hoc analyses.

*Denotes statistically significant differences compared with the standard diet (STD) group (sedentary or trained)

^Ɨ^denotes statistically significant differences compared with its untrained control group (STD-SED or HSD-SED).

STD-SED, sedentary standard chow diet; HSD-SED, sedentary high-sugar diet; STD-EXE, exercised standard chow diet; HSD-EXE, exercised high-sugar diet.

**Relative weight: [fat pad weight (g)/ body weight (g)] × 100. CS: citrate synthase.

**Table 2:** Characteristics of rats fed a STD or HSD in an 8-week period.

	8-week period				*P*-values		
	STD-SED	HSD-SED	STD-EXE	HSD-EXE	Effect of diet	Effect of exercise	Interaction
**Calorie intake (kJ)**	361.5± 44.9	327.8±87.8	413.7±66	313.9±58.8	0.1015	0.6135	0.3901
**Body weight (g)**	260.3± 14.5	235.3±14.8	210.1±17^Ɨ^	257±20.8*	0.2045	0.1057	*P*<0.001
Epididymal (g)**	0.82±0.17	1.2±0.34*	0.7±0.25	1.5±0.58*	0.045	0.5866	0.3092
Retroperitoneal (g)**	0.7±0.13	1.2±0.28*	0.4±0.19^Ɨ^	1.4±0.13*	*P*<0.001	0.7975	0.0460
Inguinal (g)**	1.4±0.27	2.4±0.84*	1.2±0.29	2.9±0.67*	*P*<0.001	0.5945	0.3091
**Adipose index**	2.9±0.5	5.2±1.45*	2.4±0.8	5.8±1.2*	*P*<0.001	0.6628	0.1790
**CS activity**	6.8±1.5	10.4±2.4	13.7±3.3^Ɨ^	10.8±2.5	0.7755	0.0036	0.088

Data are expressed as means ± S.D. Statistically significant differences were determined using a two-way ANOVA to examine the effects of diet (HSD or STD) and exercise (trained or sedentary), followed by Bonferroni post hoc analyses.

*Denotes statistically significant differences compared with the standard diet (STD) group (sedentary or trained)

^Ɨ^denotes statistically significant differences compared with its untrained control group (STD-SED or HSD-SED).

STD-SED, sedentary standard chow diet; HSD-SED, sedentary high-sugar diet; STD-EXE, exercised standard chow diet; HSD-EXE, exercised high-sugar diet.

**Relative weight: [fat pad weight (g)/ body weight (g)] × 100. CS: citrate synthase.

Regarding adipose mass, the HSD increased epididymal, retroperitoneal, and inguinal pads from the 4-week period (*P*<0.001) (Table 1). In the same way, we observed an increase in epididymal (*P*<0.05), retroperitoneal (*P*<0.001) and inguinal (*P*<0.05) depots induced by the HSD after an 8-week period, rising in HSD-SED and in HSD-EXE groups (as compared to their respective STD controls). The interaction between diet and regular physical activity decreased the retroperitoneal fat pad in the STD-EXE group (when compared to its untrained control) (Table 1). As a result, the adipose index was increased by diet (*P*<0.001) in HSD groups (sedentary or trained), raising the amount of body fat from 4-week period.

Our results showed that citrate synthase (CS) activity was influenced by diet (*P*<0.05), regular physical activity (*P*<0.001), and the interaction (*P*<0.05) in a 4-week period (Table 1), increasing its levels in STD-EXE group and decreasing in HSD-EXE groups (as compared to STD-EXE group). After an 8-week period, the effect of the HSD had disappeared, and the CS activity increased by regular physical activity and the interaction between both variables (*P<*0.05), rising in the STD-EXE group (Table 2).

### Glucometabolic parameters induced by regular physical activity and a HSD

**Fig 1:**
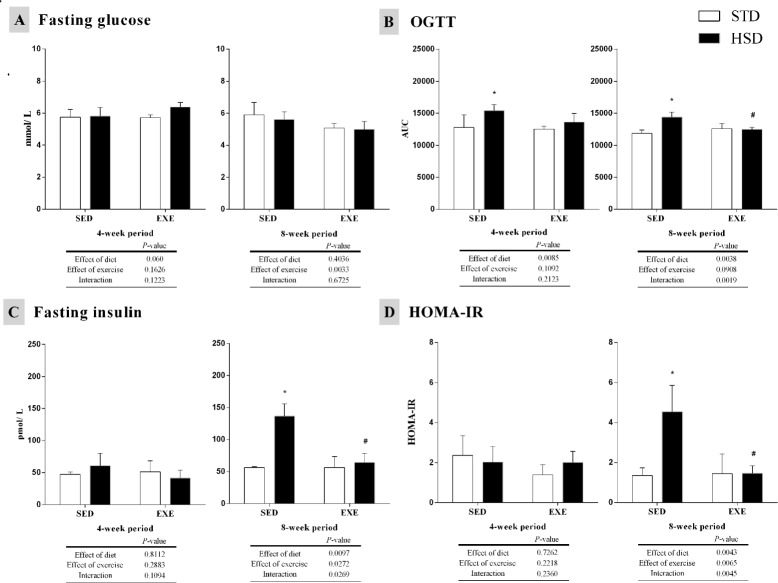
Effects of a high-sugar diet and regular physical activity (swimming without workload) on glucometabolic parameters over 4-week (left panels) and 8-week (right panels) periods. (A) Fasting glucose in plasma (mmol/L). (B) Area under the curve regarding the oral glucose tolerance test (OGTT). (C) Fasting insulin in serum (pmol/L). (D) Homeostatic model assessment index (HOMA-IR; N = 12). Data are expressed as means ± S.D. Statistically significant differences were determined using a two-way ANOVA to examine the effects of diet (HSD or STD) and regular physical activity (trained or sedentary), followed by Bonferroni post hoc analyses, *P*<0.05 was considered statistically significant. *Denotes statistical differences compared with the standard diet (STD) group (sedentary or trained), ^#^denotes statistically significant differences compared with its untrained control (STD-SED or HSD-SED). STD-SED, sedentary standard chow diet; HSD-SED, sedentary high-sugar diet; STD-EXE, exercised standard chow diet; HSD-EXE, exercised high-sugar diet.

Fig 1 summarizes the metabolic parameters analysed in this study. Glucose levels were reduced by regular physical activity after an 8-week period (*P<*0.05), although the post-hoc analysis did not show any statistically significant differences among groups (Fig 1A). In order to evaluate the metabolic response to diet sugar, we performed an OGTT, which was altered by the HSD (*P*<0.05) from a 4-week period (Fig 1B). We observed an increase in the AUC in the HSD-SED group after 4-week and 8-week periods. Regardless of the fact that the HSD impaired oral glucose tolerance, the interaction between diet and regular physical activity decreased this parameter in the HSD-EXE group (as compared to the sedentary control) after an 8-week period (P<0.05).

Insulin levels were increased by diet after an 8-week period (*P*<0.05), rising in the HSD-SED group. Furthermore, regular physical activity and the interaction were able to block the HSD effect (*P*<0.05), reducing the insulin levels of the HSD-EXE group (as compared to its sedentary control) (Fig 1C). The HOMA-IR was calculated to assess insulin resistance. Likewise to insulin levels, HOMA-IR was increased by diet after an 8-week period (*P*<0.05), rising in the HSD-SED group (as compared to STD-SED group). Regular physical activity and the interaction also blocked the HSD effect (*P*<0.05), decreasing the HOMA-IR in the HSD-EXE group (as compared to its sedentary control) (Fig 1D).

### Ultra-structural response in the gastrocnemius muscle promoted by regular physical activity and a HSD

**Fig 2:**
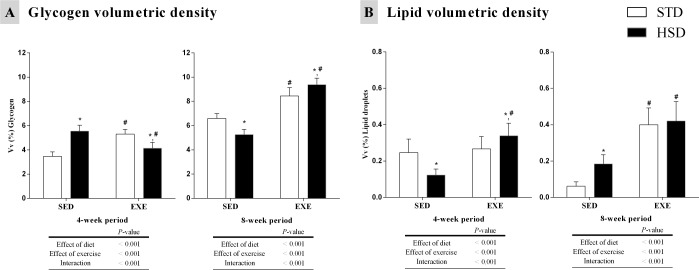
Effects of a high-sugar diet and regular physical activity (swimming without workload) on glycogen and lipids of gastrocnemius muscle fibres over 4-week and 8-week periods. (A) Quantification of glycogen and (B) lipids densities in gastrocnemius muscle fibres over 4-week (left panels) and 8-week (right panels) periods. N = 75 fields from three animals per group. Data are expressed as means ± S.D. Statistically significant differences were determined using a two-way ANOVA to examine the effects of diet (HSD or STD) and regular physical activity (trained or sedentary), followed by Bonferroni post hoc analyses; *P*<0.05 was considered statistically significant. *Denotes statistically significant differences compared with the standard diet (STD) group (sedentary or trained), ^#^denotes statistically significant differences compared with its untrained control (STD-SED or HSD-SED). STD-SED, sedentary standard chow diet; HSD-SED, sedentary high-sugar diet; STD-EXE, exercised standard chow diet; HSD-EXE, exercised high-sugar diet.

Volumetric density of glycogen and lipids in gastrocnemius muscle fibres were measured to determine muscle fuel selection induced by regular physical activity using TEM (Fig 2A and 2B). Regarding glycogen, the volumetric density was increased by diet and regular physical activity (*P<*0.001) (Fig 2A), demonstrating an increase in HSD-SED and STD-EXE groups (when compared to STD-SED group) in a 4-week period. The interaction decreased glycogen volumetric density in the HSD-EXE group (when compared to its untrained control, and when compared to STD-EXE group) after a 4-week period (*P<*0.001). After the 8-week period, glycogen density was influenced by the same parameters as the 4-week period (*P<*0.001). However, there was a decrease in the HSD-SED group as compared to the STD control (*P<*0.001), whereas regular physical activity and the interaction increased glycogen density in the STD-EXE group and in the HSD-EXE group (as compared to its untrained control and when compared to STD-EXE group) (Fig 2A).

Lipid density was reduced by diet, decreasing in the HSD-SED group (*P<*0.001). Regular physical activity and the interaction increased lipid density in the HSD-EXE group (as compared to its untrained control, and when compared to the STD-EXE group) in a 4-week period (*P<*0.001) (Fig 2B). After the 8-week period, the lipid density was influenced by the same parameters as the 4-week period (*P<*0.001). Diet increased the lipid density in the HSD-SED group, regular physical activity increased the lipid density in the STD-EXE group, whereas the interaction increased the lipid density in the HSD-EXE group (as compared STD-EXE group) (Fig 2B).

**Fig 3:**
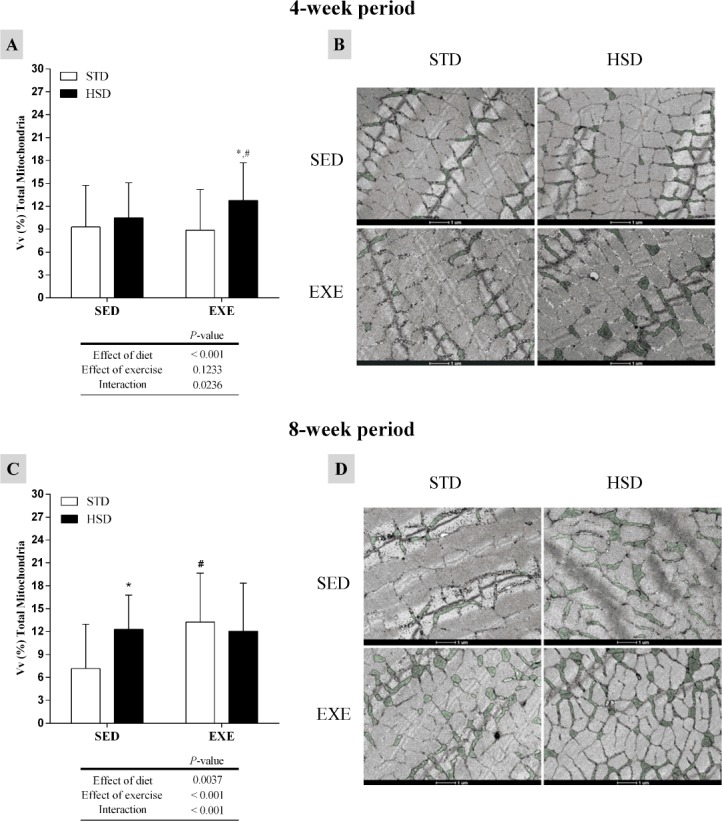
Effects of a high-sugar diet and regular physical activity (swimming without workload) on the mitochondrial profile of gastrocnemius muscle fibres over 4-week and 8-week periods. (A, C) Quantification of total mitochondria density in gastrocnemius muscle fibres (4-week and 8-week periods, respectively). N = 75 fields from three animals per group. (B, D) Transmission electron micrographs (TEM) of transverse sections of muscle fibres (ultra-structural view), in which mitochondria is highlighted in green (4-week and 8-week periods, respectively). Data are expressed as means ± S.D. Statistically significant differences were determined using a two-way ANOVA to examine the effects of diet (HSD or STD) and regular physical activity (trained or sedentary), followed by Bonferroni post hoc analyses; *P*<0.05 was considered statistically significant. *Denotes statistically significant differences compared with the standard diet (STD) group (sedentary or trained), ^#^denotes statistically significant differences compared with its untrained control (STD-SED or HSD-SED). STD-SED, sedentary standard chow diet; HSD-SED, sedentary high-sugar diet; STD-EXE, exercised standard chow diet; HSD-EXE, exercised high-sugar diet.

Total mitochondria density in the gastrocnemius muscle is shown in Fig 3. In a 4-week period, we observed the effect of the diet (*P<*0.001) and the interaction, which increased the density of total mitochondria in the HSD-EXE group, as compared to HSD-SED group and when compared to STD-EXE group (Fig 3A). After the 8-week period, the diet increased the total mitochondria density in the HSD-SED group (as compared to STD-SED) (*P<*0.05), whereas the regular physical activity and the interaction increased the total mitochondria density in the STD-EXE group, when compared to its untrained control (*P<*0.001) (Fig 3C).

**Fig 4:**
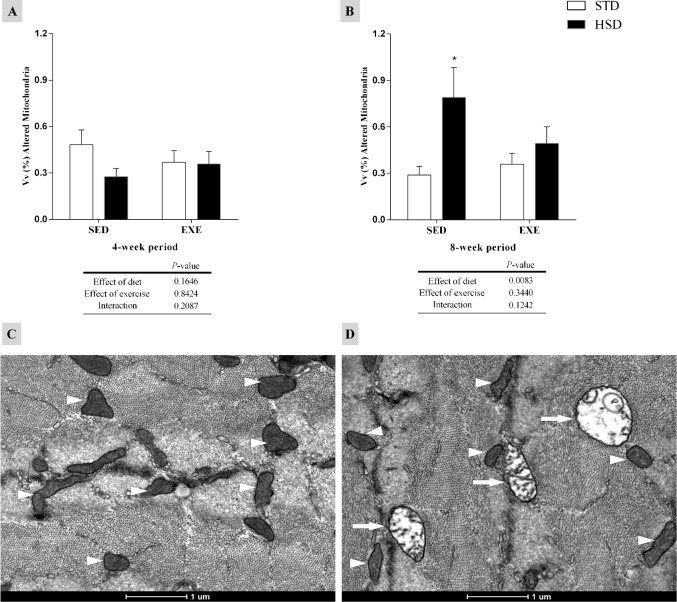
Effects of a high-sugar diet and regular physical activity (swimming without workload) on altered mitochondria in gastrocnemius muscle fibres over 4-week and 8-week periods. (A, B) Quantification of altered mitochondria density in gastrocnemius muscle fibres (4-week and 8-week periods, respectively). N = 75 fields from three animals per group. (C, D) Transmission electron micrographs (TEM) of transverse sections of muscle fibres (ultra-structural view), which represent normal mitochondria (white head arrows) from a STD rat (left panel) and altered mitochondria (white arrows) from a HSD rat (right panel), with swollen appearance, rarefied matrix and damaged cristae. Data are expressed as means ± S.D. Statistically significant differences were determined using a two-way ANOVA to examine the effects of diet (HSD or STD) and regular physical activity (trained or sedentary), followed by Bonferroni post hoc analyses; *P*<0.05 was considered statistically significant. *Denotes statistically significant differences compared with the standard diet (STD) group (sedentary or trained), ^#^denotes statistically significant differences compared with its untrained control (STD-SED or HSD-SED). STD-SED, sedentary standard chow diet; HSD-SED, sedentary high-sugar diet; STD-EXE, exercised standard chow diet; HSD-EXE, exercised high-sugar diet.

The altered mitochondria density is shown in Fig 4. These mitochondria showed a rarefied matrix and/or swollen appearance, and a disarrangement of cristae that became peripherally positioned [16, 50]. We observed an increase in the altered mitochondria density induced by the HSD (*P<*0.05) after an 8-week period (as compared to STD-SED group).

### Measurements of oxidative stress induced by regular physical activity and a HSD

**Fig 5:**
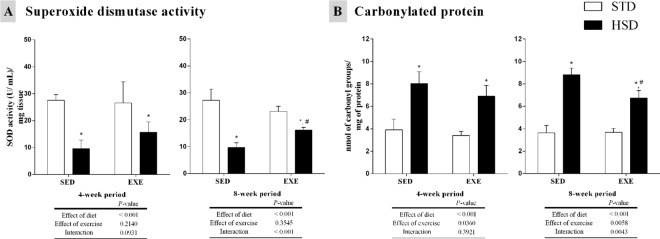
Effects of a high-sugar diet and regular physical activity (swimming without workload) on skeletal muscle oxidative stress over 4-week and 8-week periods. (A) Superoxide dismutase activity assay in gastrocnemius muscle over 4-week (left panels) and 8-week (right panels) periods. (B) Carbonyl groups per mg of protein in gastrocnemius muscle over 4-week (left panels) and 8-week (right panels) periods. N = 6. Data are expressed as means ± S.D. Statistically significant differences were determined using a two-way ANOVA to examine the effects of diet (HSD or STD) and regular physical activity (trained or sedentary), followed by Bonferroni post hoc analyses; *P*<0.05 was considered statistically significant. *Denotes statistically significant differences compared with the standard diet (STD) group (sedentary or trained), ^#^denotes statistically significant differences compared with its untrained control (STD-SED or HSD-SED). STD-SED, sedentary standard chow diet; HSD-SED, sedentary high-sugar diet; STD-EXE, exercised standard chow diet; HSD-EXE, exercised high-sugar diet.

The oxidative stress in our model was indirectly measured on two levels: accessing the antioxidant enzyme activity (SOD) and the formation of carbonyl derivatives due to protein oxidation by ROS (Fig 5) [54].

SOD activity was decreased by diet (*P<*0.001), reducing its levels in HSD-SED and HSD-EXE groups in a 4-week period. After the 8-week period, the enzyme activity was reduced by diet (*P<*0.001), decreasing in both HSD-SED and HSD-EXE groups; however, the interaction between diet and regular physical activity increased the SOD activity in the HSD-EXE group (as compared to HSD-SED group) (*P<*0.001) (Fig 5A).

Protein carbonylation was increased by diet (*P<*0.001) in HSD-SED and HSD-EXE groups in the 4-week period. After the 8-week period, the carbonyl groups per mg of protein were increased by diet (*P<*0.001), rising in both HSD-SED and HSD-EXE groups; however, regular physical activity and the interaction decreased the carbonylated proteins in the HSD-EXE group (as compared to HSD-SED group) (*P<*0.01) (Fig 5B).

### Effect of regular physical activity and a HSD on *Pgc1α* expression

**Fig 6:**
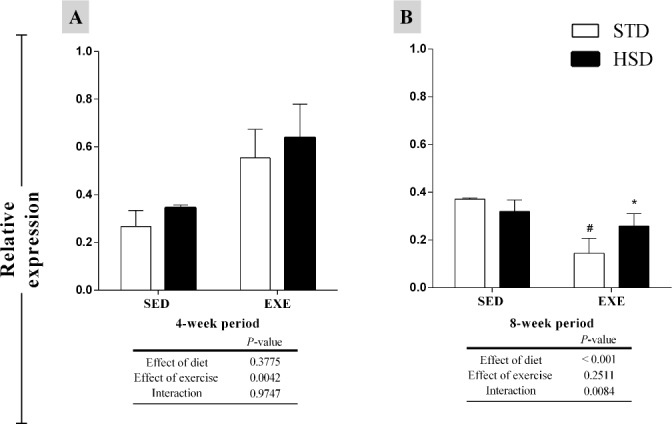
Effects of a high-sugar diet and regular physical activity (swimming without workload) on *Pgc1α* expression over 4-week and 8-week periods. (A) *Pgc1α* levels in the gastrocnemius muscle. Gene expression profiles of the groups were evaluated using the 2^-ΔCq^ method. *rRNA 18S* was used as a reference gene. Data are expressed as means ± S.D. Statistically significant differences were determined using a two-way ANOVA to examine the effects of diet (HSD or STD) and regular physical activity (trained or sedentary), followed by Bonferroni post hoc analyses; *P*<0.05 was considered statistically significant. *Denotes statistically significant differences compared with the standard diet (STD) group (sedentary or trained), ^#^denotes statistically significant differences compared with its untrained control (STD-SED or HSD-SED).

Fig 6 shows mRNA levels of *Pgc1α* in the gastrocnemius muscle. According to our results, regular physical activity had a significant effect (*P<*0.01) in *Pgc1α* expression during the 4-week period, but there were no post hoc differences (Fig 6A). After the 8-week period, both diet (*P<*0.01) and interaction (*P<*0.001) had a significant effect in *Pgc1α* expression, inducing a down regulation of ~2.6-fold in the STD-EXE group (as compared to its sedentary control); however, we also observed an ~1.8-fold increase in *Pgc1α* expression in the HSD-EXE group when as compared to STD-EXE group (Fig 6B).

## Discussion

A regular exercise program has a number of health benefits, including enhancement of cardiovascular function, muscle metabolism, mitochondrial function, and work capacity [55], in addition to preventing risk factors associated with chronic disease, thus improving overall health and lifespan [56]. However, previous data from our group have demonstrated that the association between a treadmill endurance exercise (*with* a workload) and a HSD during an 8-week period was not able to reduce the HSD-induced metabolic impairment [35,36], suggesting that the exercise type plays a crucial role the benefits it confers. In the present study, our main finding was that rats undergoing a regular physical activity (swimming *without* workload) for 8-week period resulted in an improvement on the HSD-induced IR, preventing mitochondrial morphology alterations, as well as reverting the SOD activity decreases and the carbonylated proteins increases observed in HSD sedentary group. Our results suggested a protective effect of regular physical activity in obese animals fed a HSD, preventing mitochondrial dysfunction and IR, recovering the reduction observed in *Pgc1α* induced by regular physical activity, up regulating the gene expression after an 8-week period (when compared to STD-EXE group). However, regular physical activity down regulates *Pgc1α* levels, suggesting that the mechanism behind the mitochondrial biogenesis observed in our model is not related to the PGC1α expression induced by swimming (*without* workload), in contrast to the prevalent assumption in the field.

First, although the caloric intake did not differ among groups, a HSD results in body weight increases after an 8-week period, regardless of physical activity, increasing the fat pads and the adipose index. In general, this is an expected effect of the HSD. In other words, when glucose availability increases, an insulin-induced inhibition of lipolysis occurs, resulting in decreased fatty acid oxidation and free plasma fatty acids availability [57], thus increasing fatty acids synthesis and storage. Previous results from our group have demonstrated that endurance training on a treadmill (a moderate-intensity exercise, *with* a workload) was not able to reduce the HSD-induced increase in body fat [35,36]. One plausible explanation was the lower energy efficiency in trained rats fed a HSD, which resulted in body fat accumulation due to an impairment in the relationship between *Ucp1/ Ucp3* [35]. Also, previously studies have shown that the low-intensity exercise (like ours, swimming *without* workload) was not effective in preventing neither the abdominal adiposity nor the increase in plasma and liver triglycerides associated with high-sucrose intake [58]. In addition, it was described that the relationship between exercise intensity and a reduction in visceral adipose tissue is “dose-dependent”, which means that there is a threshold for exercise intensity that determines the improvement in body composition [59]. However, regular physical activity reduced weight gain and body fat in animals fed a standard diet after an 8-week period. This result suggests that fat is the predominant source of fuel during a regular physical activity; which leads to an overall increase of the lipolysis rate, releasing fatty acids that are directly oxidized by mitochondria [60].

The adaptation of skeletal muscle oxidative capacity induced by exercise is well established and is considered a worthy indicator for exercise training efficiency [39]. Therefore, we evaluated CS activity in the soleus muscle as a parameter of endurance conditioning, only to assess if regular physical activity was able to increase the oxidative metabolism in the skeletal muscle. Along this line, CS activity levels demonstrated that regular physical activity was able to increase the aerobic capacity of rats fed a control diet (STD), which is consistent with the effect of physical activity on the oxidative metabolism in the soleus muscle [39]. On the other hand, animals fed a HSD and undergoing regular physical activity showed a reduction in CS activity levels after 4 weeks, which was lost after a 8-week period, suggesting a short effect of the HSD upon overall fat oxidation in our exercise type (swimming *without* workload) [61]. It was reported that HSD may be associated with skeletal muscle loss in older male rats [62]. Although our rats are younger, this assertive could suggest a reduction in skeletal muscle mass induced by HSD and the exercise intensity is not enough to compensate this damage at 4-week period, which reduces the CS activity in our model. After 8-week period this effect upon HSD-EXE group disappears, since skeletal muscle possibly became acclimated to a regular physical activity and the exercise might recover this loss in a small scale. In addition, the exercise type may control muscle fuel selection, increasing fatty acid oxidation while temporarily shutting down glucose oxidation, increasing muscle glucose uptake and replenishing muscle glycogen stores to prepare for the next bout of exercise [63]. This corroborates our findings, were we observe an increase in glycogen storage after an 8-week period of regular physical activity in rats fed either a STD or a HSD. We also observed an increase in the intramuscular lipid density; it has been related that the skeletal muscle from endurance-trained subjects show an increase in intramuscular lipid content, as well as an increase in the muscle oxidative capacity [64]. This phenomenon is known as the athlete’s paradox [65], since the excessive accumulation of intramuscular lipid is related to IR. However, a recent work have shown that the exercise in mice fed a high-fat diet may increase the lipid content in skeletal muscle and browns these depots, which increase the fat oxidation during exercise [66].

It is noteworthy that exercise strongly induces mitochondrial biogenesis [16–18], and PGC1α induces mitochondrial biogenesis in the skeletal muscle [19–21]; additionally, PGC1α expression in skeletal muscle is induced by exercise [67]. We observed an increase in mitochondrial density after an 8-week period of regular physical activity, which is consistent to the role of exercise inducing mitochondrial biogenesis. These results corroborate the idea that regular physical activity increases total mitochondria [16–18]. However, expression levels of *Pgc1α* were down regulated in our model during the same period, in contrast to the established statement in the field [67]. A research conducted by Perry et al. [67] demonstrated that a single bout of a high-intensity interval training (7 days) was sufficient to up regulate PGC1α expression in skeletal muscle, whereas an increase in the markers of mitochondrial biogenesis could only be observed after the third bout. Interestingly, *Pgc1α* levels returned to baseline between each bout and the induction of the gene was lessened with each bout [67]. Rowe et al. [68] demonstrated using voluntary running wheel endurance exercise and PGC1α muscle-specific knockout mice (Myo-PGC-1aKO) that *Pgc1α* is dispensable within skeletal muscle for exercise-induced mitochondrial adaptations. They also suggested that *Pgc1α* expression does not necessarily indicate a causal relationship with mitochondrial biogenesis, which corroborates our findings. Therefore, this is the first time that *Pgc1α* expression in gastrocnemius muscle is described in a model of regular physical activity (swimming *without* workload). Moreover, it is also the first time that its down regulation is correlated to an increase in total mitochondria, indicating that other pathways may exist [68]. Although further experiments might be necessary to elucidate the full mechanism related to mitochondrial biogenesis in a model of regular physical activity, it is noteworthy that the p38c mitogen-activated protein kinase (MAPK) pathway (but not p38a or b) [69] and the AMP-activated protein kinase (AMPK) have been identified as important pathways in exercise-induced mitochondrial biogenesis [70].

It is worth mentioning that the current study only evaluates mitochondrial adaptations as a result of regular physical activity, and we cannot rule out that these adaptations could result from other types of exercise, such as endurance or strength training, which might be mediated by PGC1α expression. We have chosen this type of physical activity (low-intensity swimming) due to our previously data showed that endurance training (moderate intensity exercise) does not improve metabolic parameters in rats fed a HSD [35,36]. Moreover, we used a 4-week period to determine whether this period was sufficient to detect the beginning of the changes induced by regular physical activity for the parameters assessed.

According to our results, a HSD intake during an 8-week period, results in glucose intolerance and IR, as well as in an increase in the volumetric density of mitochondria with rarefied matrix and unstructured cristae (altered mitochondria). This architectural disorganization is associated with mitochondrial dysfunction [25]. It is noteworthy that obesity may increase ROS production, which causes oxidative modifications that lead to mitochondrial dysfunction [32,33,71]. In addition, mitochondrial dysfunction has been involved in IR development, likely the result of ROS production and accumulation of by-products of lipid metabolism [14,15,72]. We evaluated ROS production indirectly by measuring the activity of the antioxidant enzyme, SOD, and the product of protein oxidation by ROS, the carbonylated protein [54]. In our study, the excessive supply of sugar increased total mitochondria, suggesting a “compensatory” increase in the mitochondrial oxidative capacity in response to dietary sugar oversupply, in a similar way that was described for rats fed a high-fat diet [73, 74]. However, our results suggest that the magnitude of these increases was not sufficient to cope with the enhanced lipid availability induced by the HSD because we observed an increase in mitochondrial morphological alterations; thus, there is still ectopic lipid accumulation and IR [73, 74]. Moreover, it is noteworthy that it is imperative for the skeletal muscle to discriminate and carefully remove damaged mitochondria [26]. This is a consequence of the interaction between PGC1α and transcription factors and nuclear receptors, including nuclear respiratory factor-1 (NRF-1) [75], NRF-2 [76], and myocyte enhancer factor 2 (MEF2) [77, 78], which are related to mitochondrial dynamics. Even though we did not conduct any experiments to evaluate mitochondrial fusion and fission processes, our results suggest that these process are impaired by a HSD once there is an accumulation of altered mitochondria [26]. Although we did not find a significant correlation between SOD activity and carbonylated proteins (unpublished data), our results showed a decrease in SOD activity and an increase in the carbonylated protein in rats fed a HSD, suggesting a role for oxidative stress in the HSD-induced mitochondrial dysfunction and IR development. More direct evidence connecting IR and mitochondrial ROS generation originated from a study by Anderson et al. [29], where the authors demonstrated that, in both rodents and humans, a high-fat diet increased the H_2_O_2_-emitting potential of mitochondria without any changes in oxidative capacity, supporting the role for oxidative stress in the development of IR [29].

Surprisingly, regular physical activity prevented the occurrence of these changes in rats fed a HSD after an 8-week period, promoting an improvement in glucometabolic parameters and preventing this architectural disorganization. Additionally, regular physical activity partially reverted the decrease in SOD activity and the increase in carbonylated proteins (which was not observed at 4-week period), suggesting a reduction in ROS production (which was indirectly measured) when compared to the sedentary control in the same period. An increase in mitochondrial function has been associated with a reduction in diabetes and obesity outcomes, and exercise has a major impact on both mitochondrial function and insulin sensitivity in skeletal muscle [18]. We observed that regular physical activity had a main effect in *Pgc1α* levels in rats fed a HSD and we also observed an increase in total mitochondria after a 4-week period. This finding is consistent with the fact that exercise is a potent up-regulator of *Pgc1α* expression, which may result in mitochondrial biogenesis [19–21, 67]. A 12-week exercise intervention program significantly increased muscle mitochondrial respiration and content in type 2 diabetes patients [79]. Similarly, an 8-week cycling exercise regime increased muscle fatty acid oxidative capacity and, in parallel, improved insulin-mediated glucose disposal [80]. Although the exercise types used were different, these findings corroborate the idea of the regular physical activity influences mitochondrial function and insulin sensitivity.

After an 8-week period, *Pgc1α* levels in trained rats fed a HSD were higher than those in trained rats fed a standard diet. This finding suggests that regular physical activity in rats fed a HSD may promote alterations in mitochondrial dynamics, conferring positive impacts on metabolic functions of skeletal muscle. In other words, although a HSD is metabolically harmful, regular physical activity can induce a functional improvement in the mitochondrial network, meaning that regular physical activity induces muscle cells to not only generate new mitochondria, but also maintain the healthy ones and remove the damaged ones [26]. Although we did not conduct any experiments to determine mitochondrial dynamics, our results suggest that regular physical activity in rats fed a HSD favours the muscle to recognize and selectively remove damaged mitochondria, thus maintaining the healthy ones, which are necessary to contain the IR in our model. Therefore, the efficient removal of altered mitochondria is critical in preserving mitochondrial function; in other words, mitochondrial biogenesis is important but it may not be the only regulatory event that leads to an improved function of the mitochondrial network [26] and insulin sensitivity in skeletal muscle in our model.

Thus, we have demonstrated that regular physical activity (swimming *without* workload) in rats fed a HSD can ameliorate the HSD-induced mitochondrial morphological alterations and glucometabolic parameters, suggesting a crucial role for regular physical activity on preserving mitochondrial function, inducing positive adaptations for metabolic homeostasis. In addition to significant changes in lifestyle, the exercise type can also suggest different molecular responses in a metabolic context, which can help us to use distinct exercise approaches to aim at different therapeutic targets.

## Supporting information

S1 Fig Representative structures analysed in the study by transmission electron microscopy. Arrows: Mitochondria irregularly shaped with sectioned tubular cristae in various planes. Arrowhead: Glycogen as electron dense granules (25–30 nm in diameter) found in inter and intramyofibrillar space as isolated particles or in clusters. LD: Lipid droplet showing homogeneous texture and no bounding membrane. Asterisks: myofibrils composed by thick filaments assembled from myosin II molecules and thin filaments from actin.
(EPS)
